# Tumor evolution and chemoresistance in ovarian cancer

**DOI:** 10.1038/s41698-018-0063-0

**Published:** 2018-09-17

**Authors:** Soochi Kim, Youngjin Han, Se Ik Kim, Hee-Seung Kim, Seong Jin Kim, Yong Sang Song

**Affiliations:** 1 Seoul National University Hospital Biomedical Research Institute, Seoul, 03080 Republic of Korea; 2 Cancer Research Institute, Seoul National University College of Medicine, Seoul, 03080 Republic of Korea; 3 WCU Biomodulation, Department of Agricultural Biotechnology, Seoul National University, Seoul, 03080 Republic of Korea; 4 Department of Obstetrics and Gynecology, Seoul National University College of Medicine, Seoul, 03080 Republic of Korea; 5 Precision Medicine Research Center, Advanced Institutes of Convergence Technology, Seoul National University, Suwon, Gyeonggi-do 16229 Republic of Korea; 6 Department of transdisciplinary Studies, Graduate School of Convergence Science and Technology, Seoul National University, Suwon, Gyeonggi-do 16229 Republic of Korea; 7 Interdisciplinary Program in Cancer Biology, Seoul National University College of Medicine, Seoul, 03080 Republic of Korea

## Abstract

Development of novel strategies to overcome chemoresistance is central goal in ovarian cancer research. Natural history of the cancer development and progression is being reconstructed by genomic datasets to understand the evolutionary pattern and direction. Recent studies suggest that intra-tumor heterogeneity (ITH) is the main cause of treatment failure by chemoresistance in many types of cancers including ovarian cancer. ITH increases the fitness of tumor to adapt to incompatible microenvironment. Understanding ITH in relation to the evolutionary pattern may result in the development of the innovative approach based on individual variability in the genetic, environment, and life style. Thus, we can reach the new big stage conquering the cancer. In this review, we will discuss the recent advances in understanding ovarian cancer biology through the use of next generation sequencing (NGS) and highlight areas of recent progress to improve precision medicine in ovarian cancer.

## Introduction

Most ovarian cancer arises from the epithelium of the ovary and fallopian tube. Cancers developing from the germ cells (eggs) or stromal cells are less common. The epithelial ovarian cancer (EOC) is not a single disease entity, rather composed of several histological subtypes, with each subtype characterized by different microscopic appearances and biological and genetic backgrounds.^1^ This diversity extends to various clinical outcomes of the disease, where patients with different histological subtypes respond differently to the same treatments and also have different prognoses. Ovarian cancer has long been classified into four representative histological subtypes, serous, endometrioid, mucinous, and clear cell adenocarcinomas.^2^ The WHO classification was recently revised and is valid since 2014.^3^ In addition, the rate of these histological subtypes of ovarian cancer are also different across racial and ethnic groups.^4^ Thus, stratification of the ovarian cancer according to their histological subtypes and tumor stage, as well as the ethnicity, are essential consideration for the decision of treatment methods.

Stage of the disease, which is determined surgically, is the critical determinant of ovarian cancer prognosis.^1^ The 5-year overall survival rate is significantly different between International Federation of Gynaecological Oncology (FIGO) stage I and stage III/IV cancers, nearly 90% and around 10–40%, respectively.^5^ There are three broad classification of prognostic factors in ovarian cancer. The tumor, the patient and lastly, the clinical interventions. The tumor itself can be sub-classified by the stage, grade, and histological subtypes. The patient with ovarian cancer are sub-classified by their age, physical, and socioeconomic status. The clinical interventions can be sub-classified by the quality of surgically removed tumor and also whether the patient have received the post-operative adjuvant chemotherapy.^5^

The standard treatment of advanced EOC is based on the maximum debulking surgery, followed by platinum-based and taxne-based chemotherapy, which remained the same over the past three decades.^6^ Meanwhile, many anticancer agents, including molecular-targeted agents, and combination therapies have been developed and validated clinically. However, the overall survival rate has not been improved significantly due to chemoresistance.^7^ Therefore, understanding the underlying molecular mechanisms associated with the chemoresistance is the critical step to improve treatment results in ovarian cancer. In this review, we will focus on the current treatment and prognosis of EOC, firstly. Next, we will explore novel strategies to overcome chemoresistance in ovarian cancer, focusing on anticancer strategy targeting tumoral evolution and intratumor heterogeneity. The recent study results of next generation sequencing (NGS) techniques will be reviewed. We will discuss the possible changes in care to pave the path towards precision medicine.

## Current treatment in EOC

In 1976, the report by Wiltshaw and Kroner on the efficacy of cisplatin in ovarian cancer opened the modern era of platinum-based combination chemotherapy.^8^ In the 1990s, Paclitaxel was introduced into platinum-based treatment, and significantly improved the progression-free survival and overall survival of the patients with advanced-stage ovarian cancer.^9^ Current standard of care for the patients with advanced-stage ovarian cancer involves primary debulking surgery followed by the platinum-based and taxane-based combination chemotherapy including Taxol and carboplatin.^6,10^ The concept of primary debulking surgery is diminishing the residual tumor to the minimum where adjuvant chemotherapy will be optimally effective.^6^ Cytoreductive surgery is initially recommended for the patients with clinical stage II–IV disease.^11^ An alternative option is neoadjuvant chemotherapy followed by interval debulking surgery, which has been shown to be safer and better tolerated than primary debulking surgery for the patients with more advanced disease.^10^ Currently, neoadjuvant chemotherapy is considered for the patients with advanced-stage disease expected in surgery or physically poor surgical candidates.^10,12–14^ However, it remains still controversial in selection of the patients, who will benefit from a neoadjuvant chemotherapy.^15,16^ Further prospective studies on this issue are warranted.

Platinum compound is the commonly selected chemo-agent for the primary treatment in ovarian cancer. Cisplatin is the very first platinum compound used as the primary treatment in ovarian cancer; however, it has a dose-limiting toxicity including minor symptoms like nausea and serious injuries on kidney and peripheral neuropathy. To overcome the toxicity associated with the use of cisplatin, the organo-platinum analogues of cisplatin, such as carboplatin has been developed and replaced cisplatin for primary chemotherapy in ovarian cancer.^17^ The following regimen was recommended by Gynecologic Cancer Inter-Group (GCIG) since 2004, and paclitaxel and carboplatin are intravenously administered every 3 weeks for six cycles in most of patients with advanced-stage ovarian cancer.^18,19^ Drug response or resistance to the chemotherapy is partly related to histological subtypes in EOC patients. High grade serous ovarian cancer patients respond well to platinum-based chemotherapy, whereas clear cells and mucinous types are remarkably platinum resistant.^20–22^ After the first round of chemotherapy, ovarian cancer patients can be sub-categorized based on the time period in which the disease progresses. NCCN guidelines in ovarian cancer defines platinum-sensitive recurrence (PSR) and platinum-resistant recurrence (PRR) based on the cut-off value of 6 months from the last day of first round chemotherapy. If patients relapse 6 months or more after the first round of platinum-based chemotherapy, they are considered PSR.^11^ Many researchers designed ovarian cancer nomogram to help both patients and clinicians to predict platinum sensitivity and overall patient survival.^23–25^ However, current limitation is that the ovarian cancer nomogram is based on data from clinicopathological parameters including age, disease stage, grade, histology, and residual disease. Future studies and development of nomograms including NGS data would improve both prediction and treatment.

Dualistic model of ovarian carcinogenesis based on morphology, molecular studies distinguishing histological subtypes of ovarian cancer by specific genetic alterations, and the mouse model of ovarian carcinogenesis attributed to our current understanding of ovarian cancer origin.^26,27^ However, the origins of ovarian cancer are still on debate, primarily because the precursor lesions of ovarian cancer are largely unknown. Fallopian tube has been suggested as origin of high-grade serous ovarian cancer (HGSOC), the most common histologic subtype of EOCs with poor outcomes. Previous studies about the prevalence of occult ovarian and fallopian tube cancers in women with *BRCA1/2* germline mutations who received risk-reducing salpingo-oophorectomy indicated the fimbriae of the fallopian tube as the potential precursor lesions of HGSOCs.^28–30^ Single cell epithelial layer with *TP53* mutation and serous tubal intraepithelial carcinoma (STIC) have been observed in patients with HGSOC.^31,32^ Adding to the immunohistochemistry, targeted sequencing also revealed that the fallopian tubal lesions harbor the same *TP53* mutations as surrounding invasive carcinomas.^33,34^ Mouse model of ovarian cancer further support this notion that HGSOCs arise from the fallopian tube.^35^ Recent evolutionary analyses using NGS technique revealed that both *TP53* mutation and STICs are precursors of ovarian cancer.^36^

### Clinico-pathological and molecular heterogeneity in ovarian cancer

**Fig. 1:**
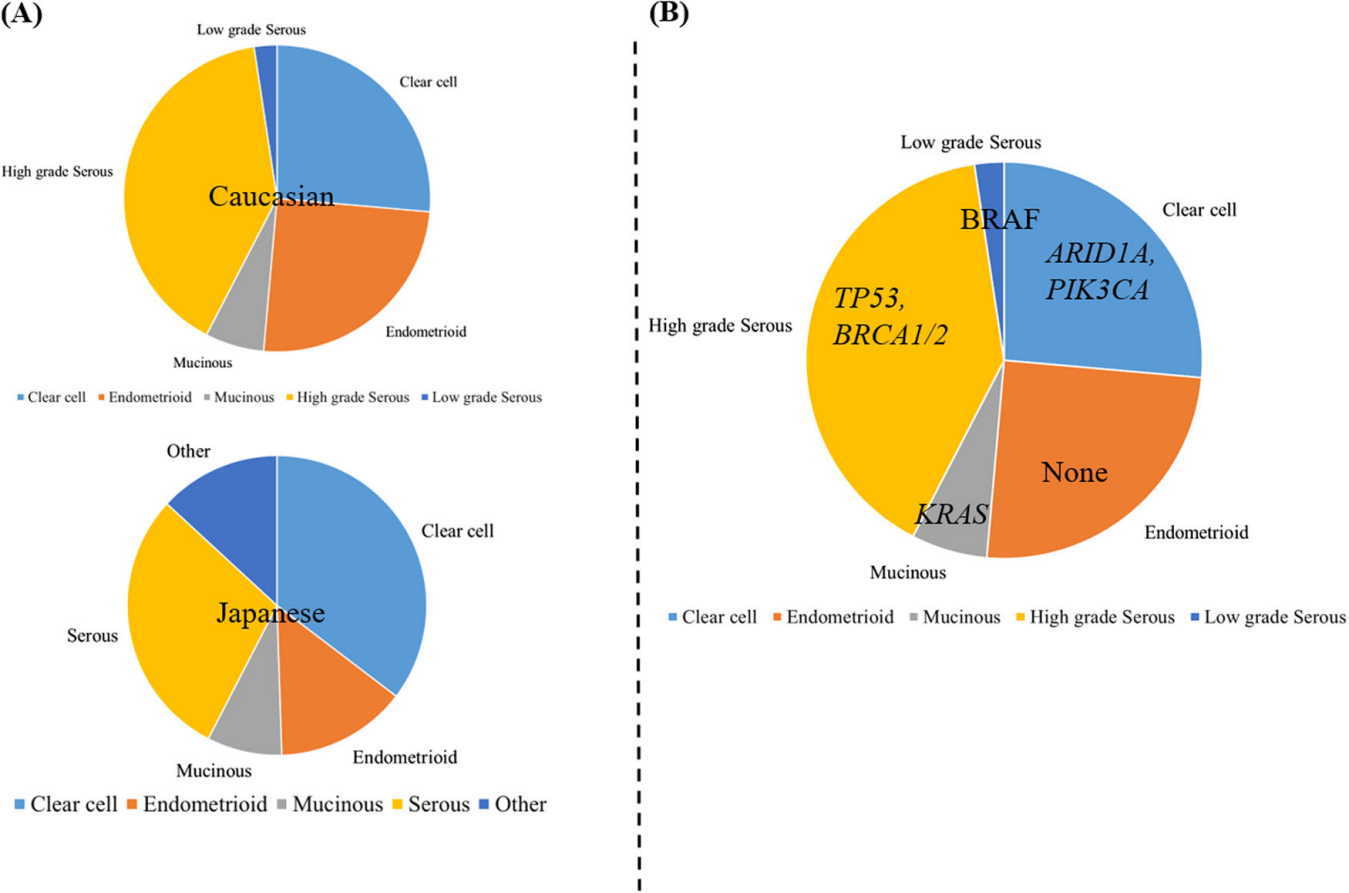
Histological and molecular heterogeneity in ovarian cancer. **a** The rate of histological subtypes in Caucasian (above) and Japanese (below). **b** Most frequently mutated genes identified by NGS technology according to histological subtypes in ovarian cancer

Kobel et al. reported clinico-pathological and molecular heterogeneity of ovarian cancer in 2008. They analyzed the expression of potential molecular markers in 500 ovarian cancer patients across all histological subtypes. Interestingly, analysis of the entire cohort identified 10 potential molecular markers which were differentially expressed between early and advanced stage ovarian cancer. However, no markers remained to be different when the analysis was restricted to each individual subtypes.^37^ The most predominant histological subtype in ovarian cancer, is serous adenocarcinoma.^4^ It has been documented that there are disparities among racial and ethnic groups with respect to the rate of histological subtypes.^38^ Particularly, the prevalence of clear cell adenocarcinoma is higher in Japan (Fig. 1a). Takenaka and his colleagues examined the clinico-pathological heterogeneity in 72 Japanese ovarian cancer patients, using targeted deep sequencing.^39^ Interestingly, the most commonly mutated genes in the entire cohort were *TP53, PIK3CA*, and *KRAS*, but there were disparities among histological subtypes with respect to the frequency of these genetic mutations. This is consistent with previous report, which demonstrated that *TP53* and *BRCA1/2* mutations are molecular and genetic signatures of serous histological subtype, and *PIK3CA* and *KRAS* are dominant mutations in clear cell and mucinous subtypes, respectively.^7,40–42^ Endometrioid histological subtype of ovarian cancer are histologically and molecularly similar to those of endometrial cancer. They share mutations in *PTEN, PIK3CA, ARID1A, KRAS, PPP2R1*, and *CTNNB1*^43–45^ but are genomically different as the frequency of *PTEN* and *CTNNB1* mutations are significantly different between the two malignancies.^46^

Recent exome sequencing study in mucinous ovarian cancer further highlighted that ovarian cancer is a heterogeneous group of tumors, and its mutation profiles are distinct across histological subtypes.^47^ This study supports that molecular features of histological subtypes are distinct (Fig. 1b) and should be considered as a group of diseases sharing the same tumor site, the ovary or fallopian tube.

Recently, our group explored the molecular heterogeneity in ovarian cancer in a number of methods. Primary culture of cellular fraction of ascites derived from ovarian cancer patients were heterogeneous and presented morphologically distinct spheres.^48^ This is consistent with data from cancer cell lines, in vivo xenograft model and patient-derived ascites.^49,50^ The clinical significance of morphologically heterogeneous tumor spheres is unclear in ovarian cancer model. However, tumor spheres have been increasingly studied in a number of cancer models, including breast cancer,^51^ glioblastoma,^52^ and pancreatic cancer.^53^ Heterogeneity in the tumor microenvironment is also known to be an important factor contributing to tumoral heterogeneity. Ascites in ovarian cancer patients created heterogeneous pro-inflammatory tumor microenvironment. At the same time, ascites increased invasiveness in a selective subset of ovarian cancer cell lines, expressing membrane-bound IL-6 receptor.^54^ In addition, pro-inflammatory M1 macrophage but not M2 macrophage increased ovarian cancer metastasis through NFκΒ activation.^55^ These findings support the presence of clinico-pathological and molecular heterogeneity in ovarian cancer caused by stromal and inflammatory cells in tumor microenvironment. Understanding clinico-pathological and molecular heterogeneity in ovarian cancer are important to improve precision medicine.^56^

### Spatial and temporal heterogeneity in ovarian cancer

Intra-tumor heterogeneity (ITH) has been documented for many decades, initially in the late 1800s by Rudolf Virchow, from a morphological aspect.^57^ The development of karyotyping and cytogenetic technologies in the 1980s and microarray technologies in the late 1990s led to numerous studies showing tumoral heterogeneity in qualitative ways. The development of NGS technologies around 2005, led to a paradigm shift in the field of genomics, away from the qualitative studies based on single markers, and to the large-scale quantitative datasets. The subsequent application of NGS technologies to human tumors revealed that ITH is common in many types of human cancers including ovarian cancer.^58–62^

NGS has been applied to the molecular characterization of tumors, and to identify new druggable targets, as well as to select appropriate patients for clinical trials.^63^ Potential druggable targets recently identified by NGS in serous and non-serous subtypes of ovarian cancer are listed in Tables 1 and 2. The lack of successful treatment strategies in ovarian cancer made the Cancer Genome Atlas (TCGA) researchers to integrate genomic analyses of ovarian cancer into molecular abnormalities related to the pathophysiology, clinical outcome, and potential druggable targets in HGSOC.^64^ The TCGA study provides a large-scale integrative view of the genomic aberrations in HGSOC with extensive heterogeneity between individual tumors. However, ITH of ovarian cancer in primary and metastatic lesions has remained largely unexplored. Recent study of our group explored the tumoral evolution during the metastasis through analysis of genetic mutations by NGS.^65^ There was a negligible accumulation of new mutations during metastasis. Interestingly, only 6% of somatic mutations were common mutations present in all samples, and the majority of somatic mutations detected in the metastatic samples were also present in the primary tumor samples. This study supports that peritoneal seeding arises with little accumulation of somatic mutations and copy number alteration, which might suggest that metastatic potential may have developed at an early stage of ovarian cancer development.

**Table 1:** Potential druggable targets identified by NGS technology in HGSOC

Samples	Druggable targets		Genomic analysis	Reference
	Dominant	Sample-specific		
Primary samples	*TP53*	*PTEN; CDKN2A*	Targeted sequencing	^39^
25 serous				
31 spatially separated samples	*TP53*	*PIK3CA; TNNB1; PDGFR; NF1; SH3GL1; RBM15*	Exome sequencing,	^58^
6 serous				
Tumor cells from ascites	*TP53*	*BRCA1; CSMD3; MACF1; CAPN7; DMD; OR5A1; PREX2; AP1b1*	Exome sequencing	^59^
3 serous				
11 spatially separated samples	*TP53*	*KIF13A; CTSG; SLCO3A1; SPIC*	Exome sequencing	^65^
1 serous				
92 serous	*TP53*	*BRCA1; BRCA2; RB1; NF1; PTEN; RAD51B*	Whole genome sequencing	^80^
Primary 80				
Ascites 12				
135 spatially and temporally separated samples	*TP53*	*BRCA1; BRCA2; APC; NF1*	Exome sequencing Targeted sequencing	^81^
4 serous				
62 temporally separated samples	*TP53*	*MECOM; ERBB2; CCNE1; ERCC2; ERCC1; TERT; BRCA1*	Exome sequencing	^82^
31 serous				
38 serous	*TP53*	*MYCC; NF1; KRAS; BRCA1*	Targeted sequencing	^112^
Primary 20				
Recurrent 18

**Table 2:** Potential druggable targets identified by NGS technology in non-serous ovarian cancer

Samples	Druggable targets		Genomic Analysis	Reference
	Dominant	Sample-specific		
Primary samples	*PIK3CA*	*KRAS; AKT1; CTNNB1; TP53*	Targeted sequencing	^39^
27 clear cell				
Primary samples	None	*PIK3CA; KRAS*;*PTEN; ERBB2; NRAS; TP53*	Targeted sequencing	^39^
10 endometrioid				
Primary samples	*ERBB2; TP53*	*PIK3CA; CDKN2A*	Targeted sequencing	^39^
3 mucinous				
Primary samples	None	*PIK3CA; PTEN; PIK3R1; ARID1A; CTNNB1*	Exome sequencing	^42^
6 endometrioid			Targeted sequencing	
Primary samples	None	*TP53, KRAS*	Exome sequencing	^42^
3 mucinous			Targeted sequencing	
Primary samples	*KRAS*	*TP53; BRAF; CDKN2A*	Exome sequencing	^47^
11 mucinous				
5 endometrioid	*KRAS*	*TP53; MYCC*	Targeted sequencing	^112^
Primary 3				
Recurrent 2				
3 clear cell	None	*TP53; BRCA1; MET*	Targeted sequencing	^112^
Primary 2				
Recurrent 1				
Primary sample	None	*TP53; AURKA; NOTCH1; FGF1R*	Targeted sequencing	^112^
1 mucinous				
Primary samples	*ARID1A*	*PIK3CA; PPP2R1A; KRAS BRAF, ERBB2, PDGFRB, PGR*	Exome sequencing	^113^
48 clear cell				
Primary samples	*PIK3CA*	*TP53, KRAS*	Targeted sequencing	^114^
105 clear cell				
Primary samples	*ARID1A; PIK3A*	*KRAS; PPP2R1A; PTEN; MLL3; ARID1B; PIK3R1*	Exome sequencing	^115, 116^
39 clear cell				

The idea that primary and metastases are clonally related is not new. In 1992, Jacob et al. performed several analyses to determine the clonal origin of metastases in ovarian cancer. This study supports that most metastases in ovarian cancer are monoclonal based on the pattern of genetic alterations including the loss of heterozygosity, p53 gene mutation and X-chromosome inactivation.^66^ Numerous studies also reported that the primary and its corresponding metastases show the same pattern of genomic alterations, which support the idea that the major gross genetic changes in ovarian cancer take place in the primary tumor.^67–70^ It has also been shown that the malignant cells presented in ascites reflect its corresponding primary tumor.^71^ Moreover, Yin and colleagues reported that synchronous bilateral ovarian cancer are monoclonal; however, primary and metastatic tumors further evolved upon dissociation.^72^

Unlike other solid tumors, the biological behavior of ovarian cancer has been thought to be unique and rather simple. Most patients are diagnosed in late-stage disease and disease is predominantly confined to the peritoneal cavity and there is a clear sign of predilected metastasis to the omentum. These clinical observations lead to a misleading conclusion in the past that the metastasis in ovarian cancer is easier and they lack hematogenous metastasis.^72,73^ Although there is a clear sign of predilected metastasis to the omentum, recent studies by Anil Sood and Buckanovich demonstrated an alternative route of metastasis in ovarian cancer, involving hematogenous pattern of metastasis in ovarian cancer.^74,75^ Using a parabiosis model, they showed that intraperitoneal injection of ovarian cancer cells in host mouse could hematogenously metastasized to the omentum of guest mouse. ErbB3-neuregulin 1 (NRG1) axis was shown to be a dominant pathway responsible for this route of metastasis.^74^ ErbB3 is overexpressed in a number of cancers including ovarian cancer and is associated with chemoresistance.^76,77^ More recently, intravenous injection of ovarian cancer cell lines generated the intra-ovarian tumor in 80% of mice and development of ascites. And also in both murine and primary human ovarian tumor cell models, subcutaneous injection of ovarian tumor cells resulted in metastasis to the ovary, supporting hematogenous spread of ovarian cancer in other metastatic sites possibly.^75^ These results support that the ovary plays a critical role in ovarian cancer cell metastasis within the peritoneal cavity.

Ovarian cancer cells leave the ovary as single cells or clusters, it is thought that they move through a passive mechanism, carried by the physiological movement of peritoneal fluid, the malignant ascites.^73^ The malignant ascites creates tumor-friendly microenvironment thereby contributing to tumor heterogeneity in ovarian cancer (reviewed in ref. ^78^). Interestingly, the degree of spatial genetic diversity varied widely across the patients, reflecting extensive intra-patient and inter-patient variability. The difference in the tumor microenvironment may explain these heterogeneity, at least in part.^58^ However, our understanding in intra-patient and inter-patient variability is limited. Larger studies are warranted to understand the pattern or direction of tumor evolution in ovarian cancer.

### Tumoral heterogeneity and chemoresistance

After cytoreductive surgery and adjuvant chemotherapy, the majority of the ovarian cancer patients achieve a clinical complete remission. However, ∼50–70% of the patients will experience the recurrent associated with occult chemoresistance. Overcoming chemoresistance is one of the major challenges faced in ovarian cancer research. ITH has been proposed as the main cause of treatment failure and drug resistance in ovarian cancer and other primary cancer.^79^

Most studies of tumor evolution and heterogeneity handled a single time-point samples, providing very indirect information. This is primarily due to difficulties in collection of longitudinal samples from cancer patients and the high costs of genomic profiling. In 2013, Bashashati et al. obtained longitudinal samples from the same patient at primary debulking surgery and after 42 months, with 21 cycles of multi-agent chemotherapy in between. Highly conserved mutations were observed between primary samples and relapsed samples, exhibiting near-identical genomic landscapes.^58^ In the same year, Castellarin et al. performed whole exome sequencing (WES) on cancer cells harvested from ascites at multiple time points. Ascites were collected at primary, at the first recurrence, and the second recurrence from three HGSOC patients receiving standard treatment to determine the clonal origin and mutational adaptations across the recurrence.^59^ From both studies, genetically distinct heterogeneous clones are considered to be present prior to treatment and most of the mutations in primary tumors persisted despite treatment, suggesting that most clones are able to evade current chemotherapy.^58,59^

In contrast, whole genome sequencing of HGSOC patients found few differences between primary tumor and ascites samples. The number of non-silent coding single nucleotide variants (SNVs) increased with the number of platinum-based chemotherapy cycles, suggesting that primary tumor further evolved during treatment. However, there were no additional platinum drug induced mutations in driver genes. Moreover, the genetic alterations were personalized rather than generalized.^80^ More recent study reported in 2015, analyzed the patterns of clonal evolution of relapsed samples in HGSOC cases using phylogenetic analysis. The phylogenetic analysis of relapsed samples showed that there were small changes in heterogeneity during neoadjuvant therapy and the prevalent clonal population at clinical recurrence arose from early divergence events.^81^ In another study, 31 paired tumor biopsies from HGSOC patient from primary debulking surgery to disease relapse were analyzed. Although the relative number of primary-specific and relapse-specific mutations vary substantially across the patients, in general, this study supported that recurrent tumors originate from a single clone that escapes platinum therapy.^82^

### Cellular heterogeneity and chemoresistance

It is well established fact that tumor tissues are comprised of diverse cellular components, including cancer cells and stromal cells.^78^ Recent advances in single-cell technology further expanded our understanding on the cellular heterogeneity by identifying cellular subsets within the tumor and stromal compartments. Proteome analysis of HGSOC at the single-cell level identified clinically relevant subsets, which were baring mesenchymal traits, based on high vimentin and low E-cadherin expression. Furthermore, these cells co-expressed cMyc and HE4, and these phenotypes were implicated in tumor development and carboplatin resistance.^83^ Epithelial to mesenchymal transition (EMT) is the feature of cancer stem cell (CSC) and implicated in ovarian cancer progression including chemoresistance. A number of molecules are associated with EMT and chemoresistance in ovarian cancer. Notch is well-known stem cell-signaling pathway, and of those the activation of Notch3 and its downstream gene SUSD2 expression is associated with EMT through upregulation of EpCAM and conferred chemoresistance to cisplatin in HGSOC.^84^ Moreover, cellular heterogeneity is more pronounced in primary tumors and cellular subsets at a transitory epithelial/mesenchymal hybrid stage that are characterized by low membrane E-cadherin, high cytoplasmic E-cadherin, high CD133, high CD44 and low Tie2 expression displayed CSC features.^85^ ^84^Other recently reported stemness and EMT regulatory genes in ovarian cancer include, KDM5A,^86^ splice isoform of CD44s (standard)^87^ and CD73.^88^ KDM5A is histone demethylase and its expression is associated with drug resistance in breast cancer^89^ glioblastoma multiform,^90^ and ovarian cancer.^86^ CD44 variant containing exons v8-10 (CD44v8-10) recently have been identified as new CSC marker in gastric cancer^91^ and cisplatin resistance in urothelial cancer.^92^ In ovarian cancer, CD44v8-10 expression in primary ovarian cancer is associated with epithelial morphology and better prognosis, whereas expression of its soluble form in ascites is associated with poor prognosis.^93^ Upregulation of transforming growth factor β (TGFβ) mediated left–right determination factor (LEFTY) was initially reported in pancreatic cancer cell lines as a novel tumor suppressor, based on suppressive effect on pancreatic cancer cell proliferation.^94^ LEFTY is identified as a new CSC marker and associated with EMT in clear cell subtype.^95^

Cellular diversity in tumor tissues and its stromal microenvironment can induce chemoresistance. Recent study showed that mesenchymal stromal cells (MSC), especially cancer-associated fibroblasts (CAFs) could induce chemoresistance in trans manner by secreting CCL2 and CCL5, which in turn promote IL-6 production in ovarian cancer cells and subsequent chemoresistance.^96^ CAFs are also main source of IL-6 secretion in ovarian tumor microenvironment and induce chemoresistance to paclitaxel.^97^ Role of IL-6 in ovarian cancer chemoresistance is noted in a number of studies and downstream molecular mechanisms implicated in chemoresistance includes PYK2,^96^ JAK2/STAT3,^97^ Ras/MEK/ERK and PI3K/Akt signaling.^98^ A heparin-binding growth factor called midkine from CAFs induced cisplatin resistance in a number of cancer cells including ovarian cancer by increasing the expression levels of lncRNA ANRIL in cancer cells.^99^ How CAFs promote chemoresistance in ovarian cancer is not fully understood yet. Recent report demonstrated crosstalk between CAFs and endothelial cells. It has been shown that CAFs regulate lipoma preferred partner (LPP) gene in endothelial cells via MFAP5 thereby increasing resistance to paclitaxel in vivo.^100^

Irrespective of germ-line and somatic mutations, BRCA1/2 mutations are known to be related with development and progression of ovarian cancer, especially for those of the high-grade serous subtype.^101–103^ Poly ADP-ribose polymerase (PARP) inhibitors, such as olaparib, rucaparib, and veliparib have recently been recognized as promising therapeutics for ovarian cancer patients with inactivating BRCA1/2 mutations as a single agent or a combination with other anticancer agents.^103–105^ To gain an insight into the selection of patients who may benefit from the treatment with PARP inhibitors, rapid and precise sequencing methods would be required. Recent study has analyzed both germline and somatic mutations in BRCA1/2 genes. The study showed detection of mutational status of BRCA1/2 by newly developed technology called ‘single-molecule molecular inversion probe (smMIP)-based targeted NGS’ using paraffin-embedded tissue.^106^ Although PARP inhibitors are effective at targeting ovarian cancer with BRCA1/2 deficiency, explained by the concept of synthetic lethality, having a good grasp of those who would benefit from the therapy is extremely important in order to potentiate therapeutic outcomes. Recent clinical trial studies have shown differential response rate of PARP inhibitor between platinum-sensitive and platinum-resistant HGSOC groups.^107^

### Tumoral heterogeneity and clinical implication (limitation)

ITH is a result of the evolutionary forces of selection pressures.^108^ During ongoing evolution, subclones are selected according to their fitness to survive in divergent microenvironmental conditions. Selection is executed based on the phenotypes or genotypes that have survival advantage in a given environment and allow further clonal expansion and evolution. Therefore, the degree of ITH in a given patient correlates with the rate of recurrent disease. This raises the question whether we could improve therapeutic responses and reduce recurrent tumor in ovarian cancer patients by alternating treatment styles.

**Fig. 2:**
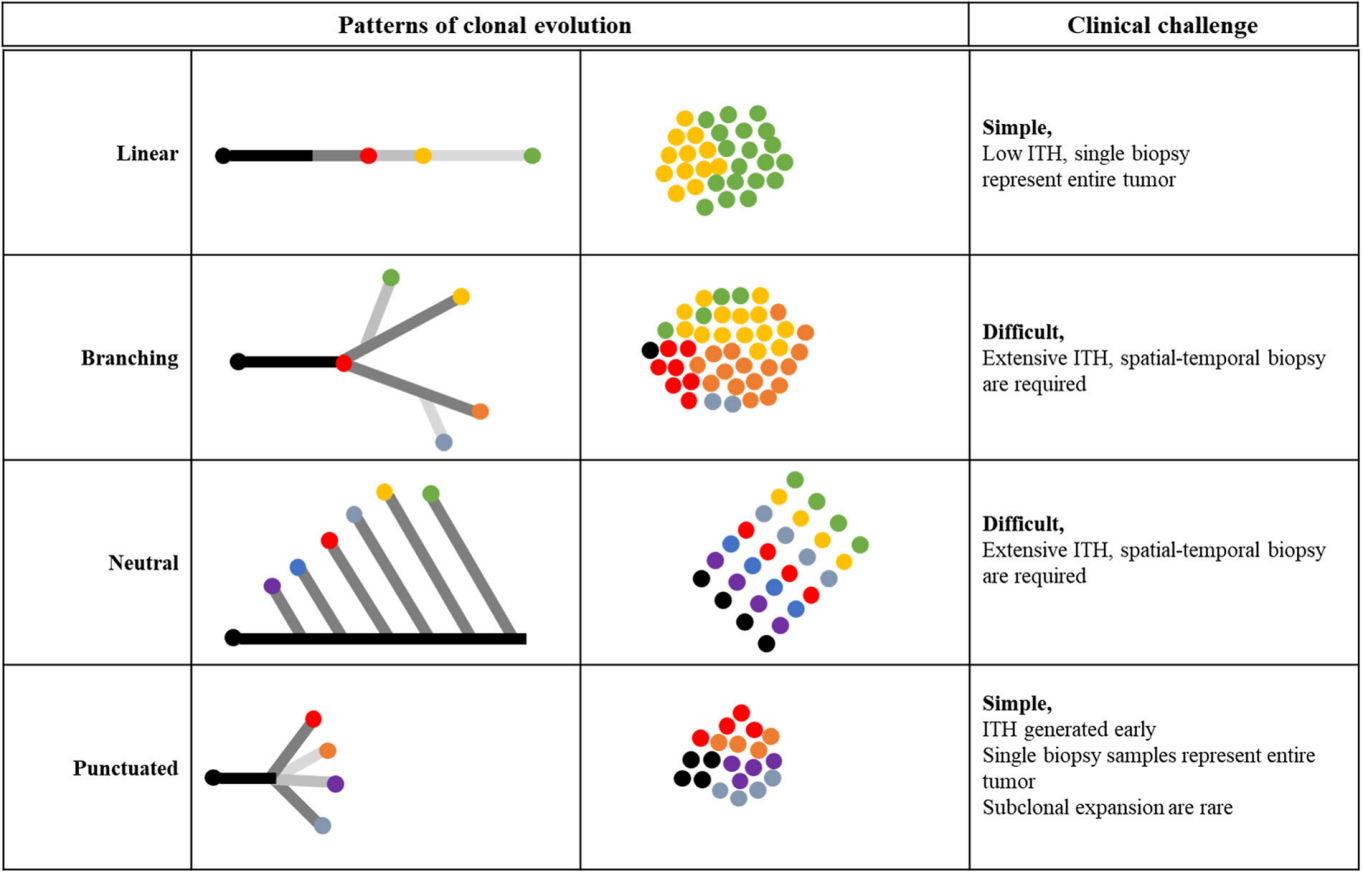
Patterns of clonal evolution and clinical challenges. Linear evolution occurs in the presence of clonal selection over time but can generate intra-tumoral and inter-tumoral heterogeneity if the selective sweep is incomplete or in a different microenvironment. Branching evolution occurs in the presence of multiple clonal selection over time, thus generate extensive ITH. Neutral evolution occurs in the absence of selective sweep but accumulation of random mutation over time result in extensive ITH. Punctuated evolution occurs in the absence of selective sweep, ITH occurs in the early stage of the tumor development and there is no further subclonal selection and expansion. Color dots indicate clones with different genotypes

NGS data can further be used to reconstruct clonal lineages and expansion to understand tumor evolution. A number of algorithms are available to construct phylogenetic trees from ITH datasets (reviewed in ref. ^109^). Thus, the history of a given tumor can be tracked retrospectively by estimating the order in which mutations occurred. There are four models of tumor evolution, linear, branching, neutral, and punctuated evolution.^110,116^ Models of tumor evolution have different implications for the clinical diagnosis, prognosis, and treatment of cancer patients (Fig. 2).

Accumulating NGS studies in ovarian cancer suggest that it is possible to improve therapeutic response and could potentially prevent the development of recurrences by targeting multiple mutated pathways present in primary tumors. Although their primary tumor is often completely removed during debulking surgery, the majority of the patients with ovarian cancer die as a consequence of metastatic disease. Studies showed a rather consistent picture of HGSOC, showing a dynamic entity composed of multiple populations of genetically and phenotypically distinct subclones evolved from a single ancestral clone with patient-specific patterns of branched evolution.^58,65,81,111^

Linear and punctuated evolution imply limited ITH, simplifying diagnostic assays, because single biopsy samples represent entire tumor. In contrast both the branching and neutral evolution suggest that ITH is extensive and would require multi-sampling approaches from different spatial regions to detect all of the clinically actionable mutations in the tumor. For example, longitudinal samples collected from primary to recurrent disease showed that a mutation which took up small portion in the primary tumor sample, became dominant close to 100% in recurrent tumor samples.^59^ These results suggest that the primary and recurrent diseases shared similar genetic alterations, but current therapy fails to destroy all the clones in the primary case. Larger studies are needed to establish whether the mutational conservation between primary and relapsed tumor samples is a feature of long survivorship in ovarian cancer patients. A number of studies support that patients with tumors with high clonal expansion show short survival and resistant relapse.^58,81^ Lambrechts et al. reconstructed tumor evolution from 31 paired surgical biopsies collected at primary and subsequent relapse. Authors failed to observe a dominant platinum-induced mutation signature but every tumor pair showed branched evolution pattern.^82^ This suggest that for HGSOC, biopsies to detect novel mutations are needed at every disease relapse for personalized anticancer therapy.

## Conclusion

The current treatment protocols for women with ovarian cancer are not subtype specific, while each histologic subtype contains unique mutation patterns. Treatment for cancer is moving towards the personalized therapy. Advances in genomics increased our understanding of the tumor genomes. We know that the cancer cells in a tumor are not all identical, composed of different clones, defined as sets of cancer cells that share a common genotype. Evolution theory successfully guided us to understand the cancer progression model. The interaction of cancer cells with microenvironment modulates tumor heterogeneity, affecting the response to therapy. Therefore, understanding tumoral heterogeneity caused by genetic alterations of cancer cells and their interaction with tumor microenvironment will help the researchers to design new therapeutic approaches towards precision medicine. Further understanding of tumor evolution with molecular, histopathological, and clinical characterizations will enlighten the precision medicine in ovarian cancer.
